# Benchmarking outcomes on multiple contextual levels in lean healthcare: a systematic review, development of a conceptual framework, and a research agenda

**DOI:** 10.1186/s12913-021-06160-6

**Published:** 2021-02-19

**Authors:** Elina Reponen, Thomas G. Rundall, Stephen M. Shortell, Janet C. Blodgett, Angelica Juarez, Ritva Jokela, Markku Mäkijärvi, Paulus Torkki

**Affiliations:** 1 Center for Lean Engagement and Research in Healthcare, School of Public Health, University of California, Berkeley, California USA; 2 HUS Helsinki University Hospital, P.O.Box 760, 00029 Helsinki, Finland; 3 Department of Public Health, University of Helsinki, Helsinki, Finland

**Keywords:** Lean management, Lean healthcare, Benchmarking, Context, Outcomes

## Abstract

**Background:**

Reliable benchmarking in Lean healthcare requires widely relevant and applicable domains for outcome metrics and careful attention to contextual levels. These levels have been poorly defined and no framework to facilitate performance benchmarking exists.

**Methods:**

We systematically searched the Pubmed, Scopus, and Web of Science databases to identify original articles reporting benchmarking on different contextual levels in Lean healthcare and critically appraised the articles. Scarcity and heterogeneity of articles prevented quantitative meta-analyses. We developed a new, widely applicable conceptual framework for benchmarking drawing on the principles of ten commonly used healthcare quality frameworks and four value statements, and suggest an agenda for future research on benchmarking in Lean healthcare.

**Results:**

We identified 22 articles on benchmarking in Lean healthcare on 4 contextual levels: intra-organizational (6 articles), regional (4), national (10), and international (2). We further categorized the articles by the domains in the proposed conceptual framework: patients (6), employed and affiliated staff (2), costs (2), and service provision (16). After critical appraisal, only one fifth of the articles were categorized as high quality.

**Conclusions:**

When making evidence-informed decisions based on current scarce literature on benchmarking in healthcare, leaders and managers should carefully consider the influence of context. The proposed conceptual framework may facilitate performance benchmarking and spreading best practices in Lean healthcare. Future research on benchmarking in Lean healthcare should include international benchmarking, defining essential factors influencing Lean initiatives on different levels of context; patient-centered benchmarking; and system-level benchmarking with a balanced set of outcomes and quality measures.

## Background

The healthcare sector worldwide is undergoing a major transformation driven by the pressures to reduce the rate of growth in healthcare spending, balance supply and demand, and improve health outcomes [1]. In industrialized countries, factors associated with increased healthcare spending include providing care to a growing aged population with chronic illnesses, incorporating technological advances, overuse and inappropriate use of care technologies, and promoting patient-centered quality of care. In the U.S. one would add the high prices charged for delivering care [2]. Additionally, inequality in access to healthcare is increasing [3].

In an attempt to address the above challenges, many healthcare organizations have adopted transformational performance improvement initiatives such as the Lean management system. Originally developed at Toyota beginning in the 1950’s, it has since spread to service industries including healthcare. In healthcare, the definition of Lean and the approach to Lean implementation are highly variable. We define Lean in healthcare as a management philosophy emphasizing patient focus, respect for people, eliminating waste and striving for excellence by engaging staff in continuous improvement and problem solving through a set of practices and tools such as A3 thinking, daily huddles, visual management, 5S (sort, set in order, shine, standardize, and sustain), and the PDSA (plan-do-study-act) cycle.

Attention to local context has been recognized as an important factor in Lean healthcare transformation sustainability, and it has been suggested that a uniform approach does not work in all contexts [4, 5]. The attributes of context are, however, often poorly defined, and current knowledge of the role of contextual factors in implementing new practices and methods such as Lean is limited [6]. Our definition of context is broad and includes all regulatory, economic, environmental, and social factors that affect the operational work of a healthcare organization. Furthermore, it is important to recognize that beyond the intra-organizational level, many contextual factors external to the organization may influence the implementation of Lean management in a healthcare organization. We identify four contextual levels: intra-organizational, regional, national, and international.

### Intra‐organizational level

The narrowest definition of context includes only intra-organizational factors such as teamwork, change resistance, ability to bridge silos, transparency, leadership commitment, communication, resources, people engagement, and empowerment [7, 8]. The intra-organizational context is rarely homogenous but rather comprises several sub-contexts in different locations and organizational units.

### Regional level

Regional factors affecting the context around Lean healthcare transformation may include geographical characteristics, market concentration and local economy, interrelations of healthcare organizations, local customs and public expectations, ethnic diversity, local authorities, and regional funding for Lean initiatives. For example, in Canada, several provinces have mandated all health regions to participate in Lean [9], and Ontario has introduced an emergency department (ED) process improvement program based on Lean principles [10].

### National level

The structures of the national healthcare system, the national healthcare funding model, and major stakeholders such as insurance companies play a major role in shaping the national landscape for Lean healthcare transformation. Furthermore, issues related to legislation and policies may influence the flexibility of task reallocation and serve as an inhibitor [11]. Labor unions and tenured staff, especially in the public sector, may hinder employee buy-in [12–15]. Variability in the cultural expectations about the roles of patients and healthcare professionals, acceptable behaviors, and the level of medicalization are other significant influencers.

### International level

There is a fourth contextual level that transcends national borders. Independent of previously described factors such as the national healthcare system model, the healthcare industry around the world has common characteristics that differentiate it from other fields such as manufacturing [16]. One limitation of implementing Lean in healthcare is that it is still relatively new in in this sector, and there is a lack of empirical evidence to convince top management of its benefits [17]. Critical breaches in the assumptions behind Lean such as the definition of the customer and limitations of capacity-led design around influencing demand or utilizing freed-up resources may emerge in the process of adopting Lean to healthcare [18]. Furthermore, some factors influencing healthcare Lean transformation are similar in all countries. For example, differences in the business logics of private and public healthcare organizations lead to different challenges in implementing Lean. The model of Lean implementation set by other service industries may be better suited to the private healthcare context [19, 20]. A tailored approach to Lean implementation may be necessary in the public sector, as public healthcare institutions are impacted by competing or even contradictory political, regulatory, and commissioning priorities [18]. Heavy bureaucracy, rigid policies, and regulations often reduce flexibility and complicate Lean implementation [13, 19]. Despite these challenges, Lean management has been shown to be feasible in the public hospital settings in many countries across the world [21–29].

### Benchmarking and Multi-Level Context

The underlying assumption of performance benchmarking is that the organizations have shared objectives represented by measurable outcomes. Following Camp, we define benchmarking as “…the search for the best industry practices which will lead to exceptional performance through the implementation of these best practices” [30]. In Lean healthcare, benchmarking could facilitate defining the best implementation strategies and practices to maximize the impact of Lean initiatives in healthcare organizations. Since most Lean healthcare organizations are still in the early stages of their Lean journey [31], benchmarking whole organizations may be neither optimal nor feasible. However, benchmarking individual quality improvement efforts may provide valuable information that helps healthcare organizations gradually reach Lean maturity. The theoretical concepts are widely shared among Lean healthcare organizations, but the practical concepts and applications are highly variable. Most healthcare organizations around the world adhere to one or several quality frameworks and value statements, many of which are compatible with Lean management philosophy. Some of the most widely recognized and adopted quality frameworks in healthcare are the Triple Aim developed by the Institute of Healthcare Improvement [32] and its modification, the Quadruple Aim [33], as well as the domains of healthcare quality defined by the National Academy of Medicine in the United States [34]. In the context of Lean healthcare, the Toyota 4P model [35] and the 10 Shingo Guiding Principles [36] are value statements adopted by many organizations. While the challenges, aims, and quality frameworks in healthcare are international, the context in which healthcare organizations in different countries and areas operate is highly variable, warranting special attention in benchmarking.

There is a call for cross-comparative research to assess possible cultural influences on Lean implementation. In their comprehensive review on Lean in healthcare, D’Andreamatteo and coworkers conclude that there are few cross-comparative and multi-site analyses, and identify the need for more research concerning different countries to allow an appreciation of the extent of using Lean in healthcare and a better evaluation of possible cultural influences [37]. No framework exists to guide such studies, and internationally relevant and applicable outcome domains are yet to be defined. Since healthcare organizations are open systems, understanding the context of Lean implementation beyond the intra-organizational level is important to reach the organization’s performance improvement goals. Cross-national comparisons would benefit early adopters and healthcare organizations in smaller countries with limited opportunities for local benchmarking and scarce research evidence directly relevant to their context. Furthermore, a better understanding of contextual differences and similarities between countries would facilitate interpreting international research findings and using them to guide a successful Lean transformation.

In this first attempt to address the challenges of cross-comparative research in Lean healthcare, we identified two equally important dimensions as prerequisites for reliable and meaningful performance benchmarking: clearly defined contextual level and a universally relevant, applicable, and balanced set of domains for benchmarking. We asked three research questions:

- On which of the context levels and outcomes has benchmarking been used in Lean healthcare?
- What outcome domains are applicable and relevant for benchmarking the performance of Lean healthcare organizations operating in different contexts?
- Based on the extent to which different contexts and outcome domains have been used to benchmark Lean initiatives, what should be the agenda for future Lean benchmarking research in healthcare?

**Fig. 1:**
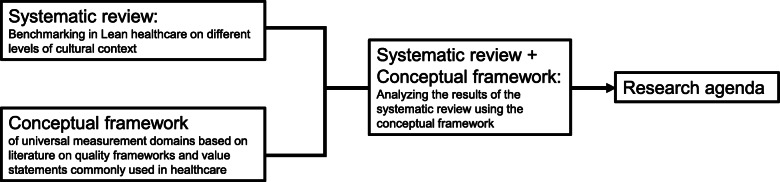
Article structure

We aim to address the first question by conducting a systematic review of current literature on benchmarking Lean in healthcare and identifying the levels of cultural context reported. Uncovering the similarities in widely used healthcare quality frameworks and value statements, we address the second question by developing a conceptual framework with a widely applicable and balanced set of outcome and quality domains and examine the articles identified through the systematic review using this novel framework. Informed by the contextual levels and outcome domains used in the articles identified through the systematic review, we identify major gaps in the existing research and propose a future research agenda that would fill those gaps and provide actionable results to the international Lean healthcare audience. Figure 1 presents the structure of this article.

## Methods

The systematic review was conducted in accordance with the Preferred Reporting Items for Systematic reviews and Meta-analyses (PRISMA) and the associated checklist was used [38]. On October 4, 2019, we conducted a literature search of English-language articles with unlimited publication years in three databases widely used for literature on healthcare management: Pubmed, Scopus, and Web of Science. The following search strategy was selected to capture all relevant articles reporting benchmarking in Lean healthcare organizations:

(Hospital OR Healthcare OR Health care) AND (Lean OR “Toyota Production System” OR “Robust Process Improvement”) AND (benchmarking OR compare OR comparison) NOT (obesity) NOT (adipose).

The terms “obesity” and “adipose” were used for excluding articles using the term “Lean” in the context of nutrition status or weight.

To be considered, the articles had to be published in English, publicly available, and peer reviewed. Furthermore, the articles had to fulfill the following predefined criteria: (1) the study was set in a hospital or healthcare context, (2) compared original data from two or more units or organizations, (3) reported using Lean methods such as those described previously in our definition of Lean, and (4) reported benchmarked outcomes in the context of Lean. The articles identified through the initial search and additional articles from reference lists went through three rounds of review: title screening, abstract review, and full text review. The review was performed by two authors (AJ and ER) independently. All disagreements were resolved through further review and discussion and, if required, with a tie-breaking vote by a third author (JB).

The methodological quality and risk of bias assessment was done at the study level for each included article using the Critical Appraisal Skills Programme (CASP) and the Specialist Unit for Review Evidence (SURE) checklists to ensure a standardized assessment across the studies [39–41]. Both CASP and SURE checklists comprise 11–12 criteria expressed as questions that are rated on a scale of yes/no/can’t tell. Examples of the questions include: Did this study address a clearly focused issue? Have the authors taken account of the potential confounding factors in the design and/or in their analysis? Are the measures of exposures and outcomes appropriate? Are the statistical methods well described? The criteria included in the CASP checklists are organized into 3 sections: validity, reporting and accuracy of the results, and generalizability of the results. In the absence of official guidance for categorizing the quality of articles based on the checklists, we defined low quality as articles meeting less than 50 % of the criteria; intermediate quality as meeting 50–74 % of the criteria, and high quality as meeting 75 % or more of the criteria on the checklists. Additionally, the studies were categorized according to the Oxford Center for Evidence-Based Medicine (CEBM) Levels of Evidence [42]. The Cochrane recommended risk of bias assessment tables for systematic reviews are designed for randomized controlled trials and not applicable for assessing other study designs. Quantitative meta-analyses were not performed due to the heterogeneity of the included studies.

Finally, we reviewed the literature to identify commonly used healthcare quality frameworks and value statements, and performed a detailed content analysis of their key elements. We then used a bottom-up approach to reveal shared domains emerging from their principles to develop a conceptual framework, which we used to further explore the articles identified through the systematic review.

## Results

### Systematic review

**Fig. 2:**
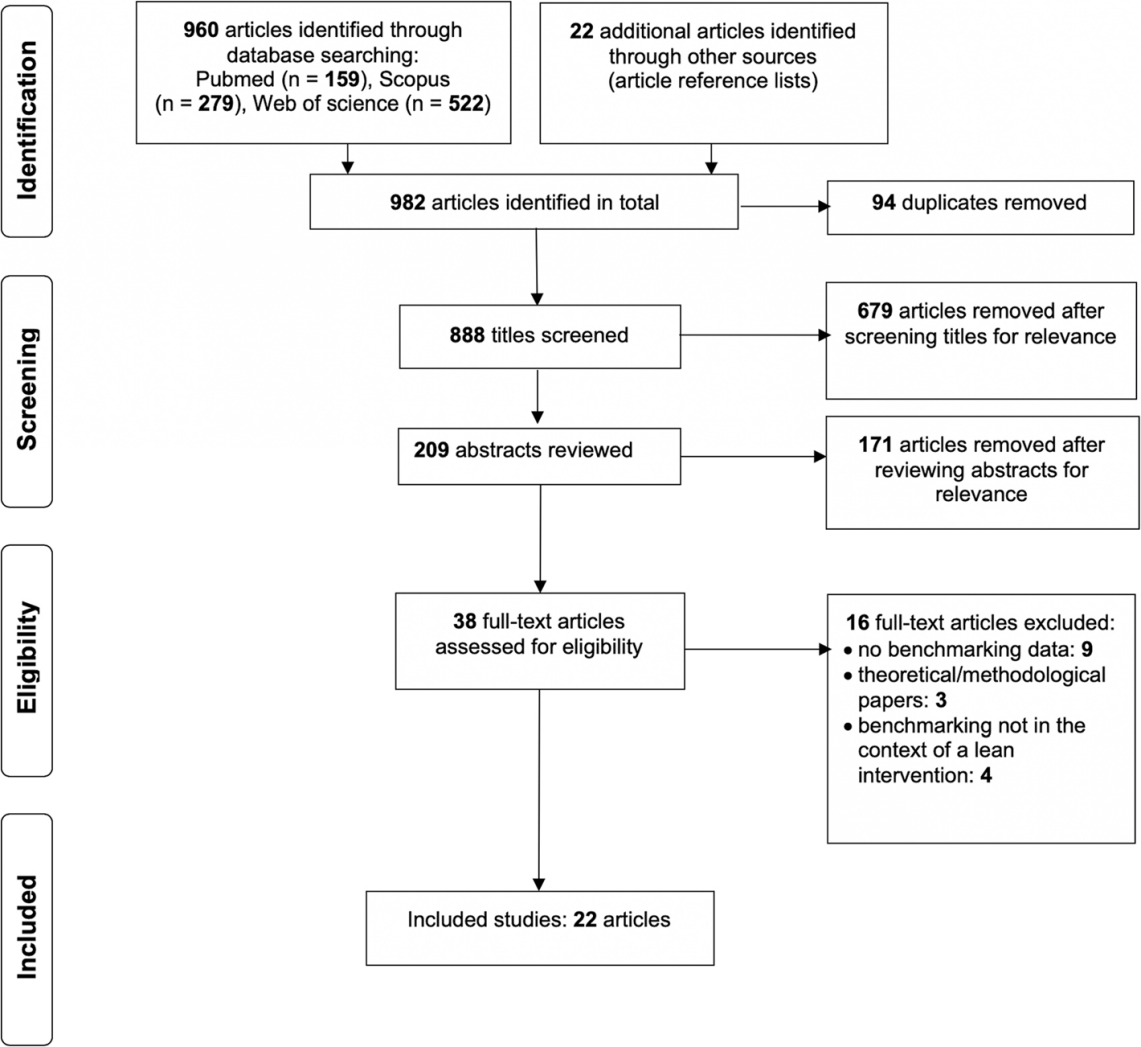
PRISMA flow diagram.

The initial search yielded a total of 960 articles: 159, 279, and 522 articles in Pubmed, Scopus, and Web of Science respectively. The authors identified an additional 22 articles through article reference lists. After removing 94 duplicates, the remaining 888 articles first went through title screening, and the abstracts of 209 articles were reviewed to determine if they met the pre-determined inclusion criteria. A total of 38 articles were selected for full text review, from which 22 articles fulfilling the criteria were identified and thus included in the final review. The two original reviewers (AJ and ER) reached agreement in all cases and no tie-breaking vote by the third author (JB) was necessary. Figure 2 presents the PRISMA flow diagram for the article selection process.

We assessed the methodological quality and risk of bias in the 22 included articles using the CASP and SURE checklists, categorizing the overall quality of the articles as low (4 articles) [43–46], intermediate (13 articles) [47–59], or high (5 articles) [31, 60–63]. All studies were cross-sectional (CEBM 4) [31, 46, 51, 54, 55], controlled interrupted time series (CEBM 3b) [47, 48, 50, 56, 60, 61, 63], or case studies (CEBM 4)[43–45, 49, 52, 53, 57–59, 62] by design. The overall quality and other detailed characteristics of the 22 studies are summarized in Table 1. The 22 articles originated in 9 different countries: Australia (1) [62], Canada (2) [45, 50], Malaysia (1) [51], The Netherlands (4) [46, 57–59], Saudi-Arabia (2) [43, 44], Spain (1) [52], Sweden (2) [54, 56], the UK (3) [59–61], and the US (9) [31, 47–49, 53, 55, 58, 59, 63], with one article reporting results from Europe without detailed information about the specific country [58]. The publication years ranged from 2008 to 2018. Six studies indicated that targeted resources were utilized in the reported Lean initiative [45, 49, 54, 55, 60, 62].

**Table 1:** Characteristics of the 22 articles included in the systematic review grouped by the level of context

Author Year	Study type/ Overall quality	Country/ region Department/ specialty	Setting for benchmarking	Benchmarking measures	Lean methods
Intra-organizational level					
Abdelhadi A [43] 2015	Multiple case study/ Low	Saudi-Arabia ED	Comparing two ED sections (male/female) within the same public hospital	•Takt time	Lean manufacturing principles to identify and eliminate waste and improve workflows
Abdelhadi A, Shakoor M [44] 2014	Multiple case study/ Low	Saudi-Arabia Pharmacy	Comparing inpatient and outpatient pharmacies at one large public regional hospital	•Takt time	Lean manufacturing principles to identify and eliminate waste and improve workflows •VSM •Spaghetti diagrams
New S et al. [60] 2016	Controlled interrupted time series/ High	UK Orthopedic OR	Comparing orthopedic trauma theater and an elective orthopedic theatre in the same trust	Primary intervention: •WHO-checklist compliance •"Glitch count” (intraoperative process disruptions) •Oxford NOTECHS II •Clinical outcomes (90D): -LOS -Complications -Readmissions Secondary intervention: •1st operation start time	Primary intervention: Lean training in •Muda •Poka-Yoke •Flow •Just-in-time •Process mapping •PDCA •Kaizen •Philosophy of continuous participative experimental improvement •Genchi Genbutsu •Respectful cooperation Secondary intervention: •Improving start time
Raab SS et al. [47] 2008	Controlled interrupted time series/ Intermediate	US/Pennsylvania Histopathology laboratory	Comparing two sister histopathology sections in one University Medical Center in Pittsburgh	•Productivity ratio (work units/FTEs)	•PPC system •A3 •Current state and ideal state identification
Robertson E et al. [61] 2015	Controlled interrupted time series/ High	UK Surgery/OR	Comparing a specialist elective orthopedic hospital’s plastic surgery team with an orthopedic theater team	•NOTECHS II (non-technical skills) •”Glitch rate” (technical skills) •WHO checklist compliance •Patient safety outcomes: -Complication rate (90D) -Readmission rate (90D) -LOS in hospital	A combination of teamwork training and lean process improvement training including: •Muda •Poka-Yoke •Genchi Genbutsu •Kaizen •Flow •Just-in-time •Respect and teamwork •Process mapping •PDCA •Philosophy of continuous improvement
Venkateswaran S et al. [48] 2013	Controlled interrupted time series/ Intermediate	US/Louisiana Hospital warehouses	Comparing three hospitals’ central warehouses in one health system	•Monthly inventory turnovers •5S audit scores (non-conformities)	Traditional 5S (control group): •Prework (5S team selection and training, baseline data collection and analysis) •Implementation (performance of 5S) •Post-analysis (evaluating outcome of the improvements) Hybrid 5S (intervention group): •Kaizen structure: -Observation and preparation (identifying problem areas, VSM) -Planning lean initiatives -Implementation (performance of first 4 S’s + developing an inventory model) -Measurement of improved process (evaluating effectiveness, efficiency, relevance, and impact)
Regional level					
Culig MH et al. [49] 2011	Case study with regional benchmarks/ Intermediate	US Cardiac surgery	Comparing results of a program with regional rates from the Society of Thoracic Surgeons National Adult Cardiac Surgery Database	•Preoperative demographics •Surgery type (off-pump, urgent, emergency, emergency salvage) •Total LOS •Post procedure LOS •Use of blood products •Complications (mortality, any complications, any infection, atrial fibrillation, cardiac arrest, heart block requiring permanent pacemaker, prolonged ventilation > 24 h, pneumonia, renal failure, reoperations, stroke, readmission within 30 days) •ICU stay •Mean total ventilation	•Vision and values •VSM •Defined metrics (balanced scorecard) •Pull methodology •Daily huddles •A3-problem solving •Ongoing mentoring of frontline staff •Visual management •Kanban •Standardization (standard work) •One-by-one processing •5S •Leveling the workload •Root cause analysis
Ieraci S et al. [62] 2008	Case study with regional benchmarks/ High	Australia/New South Wales ED	Benchmarking the ED of a single hospital against New South Wales Department of Health benchmark waiting times	•Compliance with NSW Department of Health benchmark for waiting times in each of the five Australasian Triage Scale (ATS) categories in Fast Track and Standard ED groups.	•Physical space reallocation •Creating two distinct patient tracks (low-complexity patients “fast track”, high-complexity patients “normal track”)
Kielar AZ et al.[45] 2010	Case study with regional benchmarks/ Low	Canada/Ontario Radiology	Benchmarking the performance of radiology units against provincial acceptable wait times defined by Ontario government	•Compliance with acceptable wait times for CT/MRI scans (28 days) set by the province	•Rapid Improvement Event
Vermeulen MJ et al. [50] 2014	Controlled interrupted time series/ Intermediate	Canada/Ontario ED	Benchmarking EDs in Ontario, Canada	Primary outcomes •Length of stay •Median time to physician •Percentage of admitted and nonadmitted patients missing provincial ED LOS targets Secondary outcomes •Left without being seen rate •30-day mortality •30-day readmission rate among admitted patients •72-hour revisit rate among discharged patients	A lean improvement approach, specific tools not described
National level					
Ahmed S et al. [51] 2018	Cross-sectional/ Intermediate	Malaysia Whole hospitals	Random sample of 16 hospitals in peninsular Malaysia; comparisons by respondents’ gender, type of hospital and working experience	Six Lean constructs: •Continuous quality improvement •Lean management initiatives •Six Sigma initiatives •Patient safety •Teamwork •Quality performance	Perceptions of Lean and quality improvement
Allaudeen N et al. [63] 2017	Controlled interrupted time series/ High	US ED	Benchmarking one VA ED against other similar VA facilities in the US	•ED LOS	•Root cause analysis •Developing standard work •Managing standard work: daily management system with huddles, visual management, Pareto charts, PDSA cycles
Boronat F et al. [52] 2018	Case study with national benchmarks/ Intermediate	Spain/Catalonia Urology	Comparing one Urology department with national benchmarks in Catalonia, Spain	•Risk-adjusted complications index RACI by IASIST® •Risk-adjusted mortality index RAMI by IASIST® •Risk-adjusted readmission index RARI by IASIST® •Risk-adjusted length of stay index RALOS by IASIST®	•Identification of value for the client •Identification of the value chain •Creation of continuous value flow •Elimination of the superfluous •Search for perfection by continuous improvement (PDCA) •Reduction of variability
Dickson EW et al.[53] 2009	Multiple case study/ Intermediate	US ED	Comparing four ED departments (2 academic, 2 community)	•Global patient LOS •Percentage of patients that left unseen (2/4 EDs) •Patient volume •Patient satisfaction (Press Ganey or Gallup surveys)	Kaizen events: •Current state and future state •Value stream map •Testing ideas •Continuous improvement •Pursuit of perfection
Holden RJ et al. [54] 2015	Cross-sectional/ Intermediate	Sweden Whole hospitals	Three hospitals, comparisons by hospital, unit acuity, and professional role	•Attitude toward lean •Commitment toward lean •Perceived justice of lean implementation •Perceived flow improvement due to lean	•Project-based lean implementation •Change agents and educators (internal/external)
Lee JY et al. [55] 2018	Cross-sectional/ Intermediate	US Whole hospitals	Comparing hospitals using Six Sigma vs. Lean Six Sigma in a national sample of 215 hospitals in the US	•Responsiveness capability •Patient safety •Cost	•5S •Process mapping •VSM •Kaizen •Redesign for continuous flow (cell design, pull system) •Just-in-time process management or inventory management
Pluimers DJ et al. [46] 2015	Cross-sectional/ Low	The Netherlands Colorectal cancer care pathways	Benchmarking colorectal cancer pathways in 8 hospitals	•Flowchart for rectum (yes/no) •Flowchart for colon (yes/no) •Operational focus: -Medical content, operational content, both -Mean number of patient visits •Autonomous Work Cell -Multidisciplinary outpatient clinic -Use of dedicated sessions •Physical layout -Safety, cleanness and order -Visual management system •Team -Number of staff involved with diagnosis •Pull -One stop shop for diagnosis •Non-value adding activities	•Operational focus •Autonomous work cells •Physical layout of resources •Multi-skilled teams •Pull planning •Elimination of non-value adding activities.
Poksinska BB et al. [56] 2017	Controlled interrupted time series/ Intermediate	Sweden Primary care	Comparing Lean and non-Lean groups in a national sample of health centers (primary care)	National Patient Satisfaction survey (2009, 2011, 2013), 5 subject categories: •Accessibility and waiting •Responsiveness •Patient involvement •Communication and information sharing •General impression	•Lean group (23 health centers) : at least 3 years experience working with lean •Non-lean group: no lean activities (23 health centers)
Shortell S et al. [31] 2018	Cross-sectional/ High	US Whole hospitals	Benchmarking hospitals that reported doing Lean in a national sample of US hospitals according to ownership, membership in a system or network, area type, teaching status, and bed size	•Self-reported Lean maturity •Number of years doing Lean •Number of units doing Lean •Number of tools reported as High or Very High •Overall Lean leadership commitment index •Daily management system index •Education and training scale •Self-reported performance index	A 63-item survey addressing the self-reported •Engagement in Lean, Lean Six Sigma or RPI •Duration, extent, and maturity of lean implementation •Use of tools and methods •Lean behaviors •Performance improvements
Simons P et al. [57] 2017	Case study with national benchmarks/ Intermediate	The Netherlands Oncology/radiotherapy	Benchmarking one radiotherapy institute against Dutch Society for Radiotherapy and Oncology national norms	•Percentage of patients exceeding the national norms for waiting times (palliative and curative patients)	•5S •Multidisciplinary team based projects
International level					
van Lent WAM et al. [58] 2009	Case study with international benchmarks (baseline only)/ Intermediate	The Netherlands, US, Europe Oncology	A Dutch CDU benchmarked with two other CDUs	Baseline characteristics •Patient case mix •Services offered •Total patient visits in 2004 •Estimated total patient visits in 2005 •Indexed average number of patients treated per bed per month •Indexed average number of patient visits per month per total CDU staff •Indexed average number of patient visits per nurse per month	•PDSA •Root-cause analysis •VSM •Elimination of waste •Rapid-Plan Assessment •Reorganization of inventory •Visual management
Van Vliet EJ et al. [59] 2011	Multiple case study/ Intermediate	UK, US, The Netherlands Ophthalmology	Comparing 3 cataract pathways	•Lead time •Access time •Waiting time for surgery •Number of hospital visits •Costs •Number of patients receiving their care in autonomous cataract work cells •Average number of physical patient transfers •Number of different staff functions •Number of one-stop diagnosis, preassessments, and surgeries •Number of decoupling points •Number of patients who did not receive any additional preassessments •Number of patients who did not revisit the hospital for a first review by an ophthalmologist •Number of average coordination actions per patient	•Operational focus •Autonomous work cell •Physical layout of resources •Multi-skilled team •Pull planning •Elimination of wastes

Abbreviations: *CDU* Chemotherapy Day Unit; *ED* Emergency Department; *LOS* Length of Stay; *OR* Operating Room; *PDCA* Plan-Do-Check-Act; *PDSA* Plan-Do-Study-Act; *PPC* Perfect Patient Care; *RPI* Robust Process Improvement; *VA* Department of Veterans’ Affairs; *VSM* Value Stream Mapping

We examined the 22 articles using two different categorizations: first, the level of context and second, the reported outcome domains. The most commonly used level of context was national level (10 articles) [31, 46, 51–57, 63]. All studies provided basic information on context and study setting, but there was high variation in the type and detail of contextual factors reported by the studies. A majority of the studies referred to context and culture-related issues in the discussion section, but few included an in-depth discussion of the relationship between the elements of organizational culture and the study results [60, 61]. The most frequent outcome domain in the included articles was service provision, especially process metrics [31, 43, 44, 46–50, 52, 53, 55, 58, 59, 63]. None of the studies reported outcome measures from all outcome domains in out proposed framework. Notably, regardless of the core principles of Lean described previously, Lean studies reporting outcomes related to patient experience, employed and affiliated staff, costs, and strategic perspective were scarce. The detailed results by category are presented below.

### On which of the context levels has benchmarking been used in healthcare?

#### Intra‐organizational benchmarking

We identified six studies that reported benchmarking in the context of Lean management on the intra-organizational level (Table 1) [43, 44, 47, 48, 60, 61]. Three articles benchmarked among sites that had implemented different Lean initiatives [43, 44, 48]. Three other articles benchmarked the outcomes of a Lean intervention site with non-Lean control sites [47, 60, 61], with improved process outcomes in the Lean intervention sites reported in all three articles but no significant differences in patient outcomes between the Lean intervention and control sites in two articles [60, 61].

The description of contextual factors varied across the studies. All six studies provided the geographical location (country and/or region) and general organizational setting of the study [43, 44, 47, 48, 60, 61]. However, there was little consistency in reporting other contextual factors across the six studies. While the information could be indirectly deduced from the location and hospital type, only two studies included explicit descriptions of hospital funding and governance models [43, 44]. One study included a detailed description of the national healthcare system [44]. Hospital teaching status was disclosed in two studies [44, 60]. The intervention in one study included Crew Resource Management aimed at improving teamwork and communication [61], and culture-related elements, i.e. non-technical skills, were included in the intervention and outcome measures of two studies [60, 61]. In the discussion section, three studies mentioned organizational culture and its potential influence on the results: one mentioned Lean education and development of continuous improvement culture [43]; one discussed the effect of culture on study methodology [47]; and one discussed the influence of a natural disaster, variations in operational volume, and employee cooperation and adaptability [48]. Two studies provided an in depth discussion of the relationship between elements of organizational culture and the study results [60, 61].

#### Regional benchmarking

A total of four studies reported regional-level benchmarking in Lean healthcare (Table 1) [45, 49, 50, 62]. Three of the studies reported improved outcomes after Lean implementation [45, 49, 62] whereas one study found initial benefits that seemed to diminish or disappear when benchmarked with results from control sites [50].

All four studies provided details on the location and, to a variable degree, the organizational characteristics of the study sites [45, 49, 50, 62]. and one provided an overview of the national healthcare system [45]. Two studies included elements in their intervention aimed at facilitating cultural change [49, 50]. One study discussed the mechanisms and role of culture change, including a “no blame” culture and empowerment of staff [49]. whereas another identified the lack of measuring contextual factors such as management involvement and staff buy-in as a limitation.[50] Two studies did not discuss the role of contextual factors.[45, 62].

#### National benchmarking

Ten studies used benchmarking in Lean healthcare on a national level (Table 1) [31, 46, 51–57, 63]. Two studies found Lean implementation improved performance against national benchmarks [52, 57], and another three studies reported improved outcomes after the implementation of Lean initiatives [53, 55, 63].

All ten studies defined the location [51–54, 63] and, with the exception of one study [46], all provided some organizational characteristics of study sites, albeit with a varying degree of detail. Five studies reported the teaching status or academic affiliation, or the lack thereof, of study sites [31, 53–55, 63], and the ownership (public or private) was explicitly stated in five studies [31, 51, 52, 54, 55]. With the exception of the study utilizing patient satisfaction survey data [56], all survey-based studies included some questions related to organizational culture [31, 51, 54, 55]. Two studies included elements targeting staff buy-in and cultural change in the intervention [52, 57]. Measures related to organizational culture, i.e. safety culture, employee satisfaction, and absenteeism, were used as outcomes in one study [57].

All ten studies referred to culture-related and contextual issues in the discussion. Five studies identified the general associations of organizational culture and context with outcomes [31, 51, 55, 57, 63]. Specific cultural context elements identified as important contributing factors were team training and feedback improvement [52], the importance of adapting the Lean approach to local culture, [53] and the influence of culture and context such as leadership support on outcomes [53]. Two studies acknowledged that the partial knowledge of context factors was a limitation of the study [46, 56]. One study outlined three levels of context: unit/role/team, regional/hospital, and national level [54], but issues beyond the organizational culture, particularly the influence of the local national healthcare system were discussed in only one study [52].

#### International benchmarking

Only two studies reported benchmarking in Lean healthcare on the international level (Table 1) [58, 59]. One used benchmarking to compare performance levels and operational differences in three organizations with the results guiding the design of a Lean process improvement intervention in one of the organizations, but provided little contextual information besides the geographical location about the benchmarking sites nor discussion of the role and impact of contextual factors [58].

The other study benchmarked the operations of three Lean eye hospitals in the UK, the US and the Netherlands, addressing six Lean aspects [59]. The authors concluded that the operational focus of the participating hospitals was influenced by external contextual factors leading to different objectives. This study provided details on the location, type, teaching status, and operational volume of the organizations. In the discussion, the authors identified the effect of environmental context on how Lean was applied and the role of organizational culture in Lean implementation. Furthermore, the authors identified the study methodology as a limiting factor for the assessment of the effects of contextual factors.

### What outcome domains have been used within each context level?

#### Conceptual framework for the selection of outcome and quality measures to facilitate benchmarking

The above review revealed a wide variation and a lack of consistency in the selection of outcome measures among the benchmarking studies. To address this issue, we integrated the overarching themes of 10 quality frameworks [32] and four value statements [35, 36, 71, 72] into a single framework with four main domains: patients, employed and affiliated staff, costs, and service provision. The main domain of patients comprises two subdomains: clinical outcome and experience. The service provision domain includes four subdomains: access, processes, continuous improvement, and strategic perspective. Table 2 shows the relevance of these key domains regardless of the framework or value statement chosen by an individual healthcare organization highlighting the applicability of these domains in Lean healthcare organizations despite the variability in the definition of and approach to Lean.

**Table 2:** Proposed conceptual framework dimensions based on key elements of healthcare quality frameworks and value statements

	Patients		Employed and affiliated staff	Costs	Service provision			
	**Clinical outcome**	**Experience**			**Access**	**Processes**	**Continuous improvement**	**Strategic perspective**
***Quality frameworks***								
**IHI Triple aim **[32]	• Population health	• Experience of care		• Per capita cost				
**Quadruple aim **[33]	• Population health	• Experience of care	• Wellbeing	• Per capita cost				
**IOM domains of healthcare quality** [34]	• Safe • Effective	• Patient-centered		• Efficient	• Timely • Equitable			
**Balanced scorecard**: **Perspectives **[64]	• Customer value	• Customer satisfaction and/or retention	• Learning & growth o human capital o culture	• Financial o financial performance o effective resource use		• Internal process o efficiency o quality		• Learning & growth o infrastructure & technology o culture
**The Malcolm Baldrige Criteria for Performance Excellence **[65]	• Results: o Product/Service	• Results: o Customer Satisfaction	• Workforce focus • Results: workforce	• Results: Financial and marketplace performance		• Results: operational effectiveness	• Measurement, analysis, and knowledge management	• Leadership • Strategic planning • Results: Leadership
	• Customer Focus							
**OECD Health Care Quality Indicators Framework **[66]	• Quality: o Effectiveness o Safety	• Quality: o Responsiveness o Patient Centeredness		• Cost/Expenditure	• Access o Accessibility			
**Total Quality Management Principles **[67]		• Customer Satisfaction	• Employee commitment				• Continuous Improvement	• Fact-Based Decision Making • Effective Communications • Strategic Thinking • Integrated System
**WHO Regional Office for Europe: Performance Assessment Tool for Quality Improvement in Hospitals (PATH) **[68]	• Clinical effectiveness • Safety	• Patient Centeredness	• Staff orientation			• Production efficiency		• Responsive Governance
**Campbell et al.** **Quality of Care Conceptual Framework **[69]	• Effectiveness: o Effectiveness of Clinical care	• Effectiveness: o Effectiveness of inter-personal care			• Accessibility: o Affordability o Availability			
**A framework for High-Reliability Organizations in Healthcare **[70]		• Engagement of patients and family	• Culture of safety o Psychological safety o Accountability o Teamwork & communication o Negotiation				• Learning system o Continuous learning o Improvement & measurement o Reliability o Transparency	• Effective leadership
***Value statements***								
**WHO: Goals of the Health System**[71]	• Optimal health for all	• Responsiveness of care provision system		• Fair financing				
**Shingo guiding principles**[36]	• Assure quality at the source	• Respect every individual	• Respect every individual • Create constancy of purpose			• Focus on process • Flow & pull value	• Seek Perfection • Embrace Scientific thinking	• Lead with humility • Think systemically
	• Create value for the customer							
**Lean 4P Model**[35]	• Philosophy (Create value, customer focus)		• People			• Process	• Problem solving	
**EFQM Excellence Model: Fundamental Concepts of Excellence**[72]	• Adding Value for Customers					• Managing with agility	• Creating a sustainable future • Developing organizational capability • Harnessing creativity and innovation	• Leading with Vision, Inspiration and Integrity

Abbreviations: *EFQM* the European Foundation for Quality Management; *IHI* Institute for Healthcare Improvement; *IOM* Institute of Medicine; *OECD* Organisation for Economic Co-operation and Development; *WHO* World Health Organization

We classified the 22 articles identified through the systematic review according to the benchmarked outcomes using the proposed conceptual framework and the four levels of context identified through the literature (Table 3). Unsurprisingly, the most frequent main domain among the 22 articles was service provision, which was a focus in 17 articles [31, 43–50, 52, 53, 55, 57–59, 62, 63]. Of these 17 articles, 14 used outcome measures related to processes [31, 43, 44, 46–50, 52, 53, 55, 58, 59, 63], and five articles used outcome measures related to access to care [45, 50, 57, 59, 62]. Clinical outcomes were benchmarked in seven articles [31, 49, 50, 52, 55, 60, 61], and patient experience in two articles.[53, 56] Factors related to employed and affiliated staff were benchmarked in three [31, 51, 54] and costs in two articles [55, 59]. Only one article [31] benchmarked outcomes related to continuous improvement or strategic perspective, both subdomains under the main outcome domain of service provision.

**Table 3:** Classification of the articles included in the systematic review using the proposed conceptual framework

Context level	Article	Patients		Employed and affiliated staff	Costs	Service provision			
		**Clinical outcome**	**Experience**			**Access**	**Processes**	**Continuous improvement**	**Strategic perspective**
Intra-organizational	Abdelhadi A[43]						**X**		
	Abdelhadi A, Shakoor M[44]						**X**		
	New S et al .[60]	**X**							
	Raab SS et al .[47]						**X**		
	Robertson E et al .[61]	**X**							
	Venkateswaran S et al .[48]						**X**		
Regional	Culig MH et al .[49]	**X**					**X**		
	Ieraci S et al .[62]					**X**			
	Kielar AZ et al .[45]					**X**			
	Vermeulen MJ et al .[50]	**X**				**X**	**X**		
National	Ahmed S et al .[51]			**X**					
	Allaudeen N et al .[63]						**X**		
	Boronat F et al .[52]	**X**					**X**		
	Dickson EW et al .[53]		**X**				**X**		
	Holden RJ et al .[54]			**X**					
	Lee JY et al .[55]	**X**			**X**		**X**		
	Pluimers DJ et al .[46]						**X**		
	Poksinska BB et al .[56]		**X**						
	Shortell et al .[31]	**X**		**X**			**X**	**X**	**X**
	Simons P et al .[57]					**X**			
International	van Lent WAM et al .[58]						**X**		
	Van Vliet EJ et al .[59]				**X**	**X**	**X**		

#### Patients: clinical outcome

In the critical appraisal, four of the seven articles that benchmarked clinical outcomes were categorized as intermediate [49, 50, 52, 55] and three as high overall quality [31, 60, 61]. Two articles represented benchmarking on intra-organizational, [60, 61] two on regional, [49, 50] and three on national level of context [31, 52, 55]. Three studies indicated a positive effect of Lean implementation on patient outcomes, [49, 52, 55] whereas three studies failed to demonstrate a positive effect [50, 60, 61]. One study used a self-reported performance index that included, among other metrics, measures related to patient outcomes such as reducing medical errors [31].

#### Patients: experience

The two studies that benchmarked patient experience were both categorized as intermediate overall quality and represented the national level of context [53, 56]. However, their results were contradictory: some Lean initiatives were associated with improved patient satisfaction whereas others were not.

#### Employed and affiliated staff

Two studies using the employee perspectives on Lean for benchmarking were categorized as intermediate [51, 54] and one as high overall quality [31]. All three represented the national level of context. None of the studies used non-Lean controls: the studies benchmarked employees’ views or education and training on Lean in different types of institutions and roles.

#### Costs

One study benchmarked the costs on the national level of context [55] and one study on the international level of context [59]. Both were categorized as intermediate overall quality. Both studies indicated a possible cost-saving effect with Lean implementation.

#### Service provision: access

Of the five studies benchmarking access, one was categorized as low [45] ,three as intermediate [50, 57, 59], and one as high overall quality [62]. Three articles benchmarked access on the regional level, [45, 50, 62] one on the national level [57], and one on the international level of context [59]. Three of the five studies indicated that Lean implementation positively affected access [45, 57, 62], whereas the fourth concluded no significant difference compared to control sites [50], and the fifth study did not have a non-Lean comparison [59].

#### Service provision: processes

There were a total of 14 articles that benchmarked process measures. Nine were categorized as intermediate [47–50, 52, 53, 55, 58, 59], three as low [43, 44, 46], and two as high overall quality [31, 63]. Four studies benchmarked processes on the intra-organizational level [43, 44, 47, 48], two on the regional level [49, 50], six on the national level [31, 46, 52, 53, 55, 63], and two on the international level of context [58, 59]. A majority of the study designs did not include a non-Lean comparison [31, 43, 44, 46, 48, 53, 58, 59]. Five studies reported that Lean implementation had predominantly positive effects on process metrics,[47, 49, 52, 55, 63] whereas in one study a difference-in-differences analyses indicated no benefit for the Lean sites when compared to non-Lean control sites [50].

#### Service provision: continuous improvement and strategic perspective

Only one study used benchmarking measures related to continuous improvement represented by the daily management system index, and strategic perspective represented by the Lean leadership commitment index, both subdomains of service provision.[31] The overall quality of this article was high, but it did not use non-Lean controls for benchmarking.

## DISCUSSION

Lean is a set of organizational principles, practices, and problem-solving tools designed for improving quality and processes. The existing literature on benchmarking in Lean healthcare is surprisingly scarce considering the relatively widespread adoption of Lean in healthcare organizations, and is dominated by results from the US much like Lean-related literature in healthcare in general [17]. Furthermore, there is need for improved quality of the research in the area: after critical appraisal, only 22.7 % of the studies were categorized as high overall quality. These findings are consistent with previous systematic reviews that have criticized existing literature on Lean healthcare for the lack of rigorous methodology [73, 74]. Since Lean has gained popularity in healthcare during the last 15–20 years, the research in this field is still young: all studies included in our systematic review are published in or after 2008. Unsurprisingly, a majority of the included studies focused on benchmarking process metrics, perhaps reflecting the manufacturing origins of Lean tools and methods [75]. Perhaps due to the heterogeneity and relatively low number of articles included in this systematic review, we could not identify any trends in the sustainability of Lean strategies and initiatives over the 10-year period during which the articles were published. Furthermore, benchmarking in Lean healthcare has yet to truly transcend international borders. While many general elements such as patient focus are widely adopted by healthcare organizations implementing Lean, the lack of consensus on the definition of Lean and the highly variable approaches different organizations have taken on their Lean journey may further complicate comparative research in the field.

The context is an important factor to consider in healthcare Lean transformation. Each healthcare organization is inevitably influenced by factors on all four levels of context, and these factors should be recognized and addressed when benchmarking is used; the greater the geographic distance between the benchmarked organizations, the more complex the differences in the context. Identifying the levels of context facilitates a comprehensive approach to help with better understanding the validity of the benchmarking results.

No consensus on the dimensions of performance measurement and benchmarking in Lean healthcare exists. Our proposed conceptual framework identifies the outcome domains based on the values and quality frameworks shared by most healthcare organizations to guide measuring performance and quality in Lean healthcare and facilitate benchmarking. Additionally, the framework could facilitate establishing a balanced set of benchmarking measures reflecting all outcome domains for each level of context.

For leaders and managers our findings suggest that there is some benchmarking research that identifies contextual factors affecting Lean performance that they can use in making decisions about Lean adoption and implementation. But that research is generally limited both in terms of the levels of context addressed in any given study and the types of performance outcomes for which any context is reported. Hence, caution and in-house assessments of contextual factors and their possible effects on Lean will be important.

For researchers, our findings reveal gaps in current research that should be addressed in future studies to increase the likelihood that decisions about Lean adoption and implementation will be better informed with evidence about the potential effects of context. Based on our findings, we suggest the following directions for a future research agenda:

- *Research on international level benchmarking in Lean healthcare*.

Categorizing the included articles by the level of context indicates that despite the growing interest in transformational performance improvement among nearly all countries, benchmarking has rarely been used beyond the national level. Only two of the studies reported international benchmarking, both in distinct clinical subspecialties. The worldwide use of Lean methodology to transform healthcare highlights the need to address the complexities of international benchmarking to expand knowledge in the field.

(b) *Essential factors on different levels of context influencing the results of Lean initiatives*.

The characteristics of the context reported in existing studies are highly variable and the influence of contextual factors beyond the intra-organizational level was discussed in only one study. Less than one third of the included articles indicated additional resources allocated to the Lean initiative, yet their potential impact on the results was not discussed in depth. Thus, further work is necessary to identify the most essential characteristics of context to enhance the generalizability and applicability of benchmarking results to other countries, regions, and organizations.

(c) *Patient-centered benchmarking in Lean healthcare*.

The previously recognized need to tie Lean process improvement efforts to the ultimate goals of healthcare [8] is also evident in our results: patient outcomes were the second most frequent performance domain benchmarked in the studies included in our systematic review. Patient experience, however, was only measured in two studies both on the national level of context indicating an important future direction for patient-centered benchmarking on multiple contextual levels.

(d) *System level research using a balanced set of outcome and quality measures*.

The large number of studies using benchmarking measures primarily reflecting processes compared to studies using benchmarking measures reflecting access may also be an indicator of the low maturity of Lean implementation in the healthcare sector. The focus is still primarily on production and intra-organizational processes whereas fewer studies have taken a broader perspective on service provision at the system level beyond the scope of a single organization. Time is the single most frequently used measure for benchmarking in Lean healthcare. Time, while easy to measure and an indicator of patient flow and throughput, cannot adequately measure costs or the quality of care. For a more balanced approach, some of the studies used additional measures such as readmission rates. Most of the studies reported benchmarking measures from only one or two outcome domains. None of the studies used measures from all four main domains and, in particular, studies on benchmarking the subdomains of continuous improvement or strategic perspective are rare, highlighting the need for future studies with a balanced set of benchmarking measures.

### Strengths and limitations

Our systematic review has two main strengths. First, it is based on relatively broad literature search criteria to increase the likelihood of capturing relevant articles. Second, our pre-defined inclusion criteria intentionally allowed a range of study designs, providing as comprehensive an understanding of the existing literature as possible. Furthermore, we conducted a critical appraisal of all included studies and indicate the results in the review text and tables, but did not exclude any articles from the review even if the overall quality was categorized as low.

This systematic review also has limitations. Despite the broad search criteria, we may have missed some articles that used some other terms for benchmarking. To decrease the likelihood, we added the words “compare” and “comparison” to the search strategy. We also cannot discount publication bias, which may have influenced the results of our systematic review. Due to the broad search and inclusion criteria the study designs and outcomes were highly variable. Together with the low overall number of studies, this prevented a meta-analysis of the results.

## Conclusions

Lean empowers frontline staff to eliminate waste and to continuously improve through standard work and problem solving. Studies on benchmarking in Lean healthcare are scarce and mostly limited to intra-organizational, regional, and national levels of context. The most commonly used benchmarking measures represent the domain of service provision, particularly process outcome metrics, and studies with fully balanced sets of benchmarking measures are lacking. Leaders and managers should pay careful attention to the limited extent of information on contextual factors when making evidence-informed decisions based on current Lean healthcare benchmarking literature. The proposed conceptual framework defining the outcome domains emerging from widely used quality frameworks and value statements may facilitate performance benchmarking and spreading best practices in Lean healthcare. Future research in Lean healthcare benchmarking should include international benchmarking, defining essential factors influencing Lean initiatives on different levels of context, patient-centered benchmarking, and system-level benchmarking with a balanced set of outcomes and quality measures.

## Data Availability

All data used in the systematic review were obtained from publicly available internet databases (Pubmed, Scopus, and Web of Science). The datasets used during the current study are available from the corresponding author on reasonable request.

## List of Abbreviations

CASP: Critical Appraisal Skills Programme
CDU: Chemotherapy Day Unit
CEBM: Center for Evidence-Based Medicine
ED: Emergency department
EFQM: the European Foundation for Quality Management
IHI: Institute for Healthcare Improvement
IOM: Insititute of Medicine
LOS: Length of Stay
OECD: Organization for Economic Co-operation and Development
OR: Operating Room
PDCA: Plan-do-check-act
PDSA: Plan-do-study-act
PPC: Perfect Patient Care
PRISMA: Preferred Reporting Items for Systematic Reviews and Meta-analyses
RPI: Robust Process Improvement
SURE: Specialist Unit for Review Evidence
VA: Department of Veterans’ Affairs
VSM: Value Stream Mapping
WHO: World Health Organization
5S: Lean tool, short for ”sort, set in order, shine, standardize, and sustain”
